# 4-Chloro­phenyl 2-oxo-2*H*-chromene-3-carboxyl­ate

**DOI:** 10.1107/S1600536812003376

**Published:** 2012-02-04

**Authors:** Xiao-Qiang Guo, Jun Yan, Ya Gan, Qin Song, Xiao-Jun Gou

**Affiliations:** a Faculty of Biotechnology Industry, Chengdu University, Chengdu 610106, People’s Republic of China

## Abstract

In title compound, C_16_H_9_ClO_4_, the coumarin ring system is approximately planar [maximum deviation = 0.056 (1) Å] and is oriented with respect to the benzene ring at an angle of 22.60 (7)°. Inter­molecular C—H⋯O hydrogen bonding is present in the crystal.

## Related literature

For the biochemical properties of related compounds, see: Kontogiorgis & Hadjipavlou-Litina (2005 ▶); Finn *et al.* (2002 ▶); Gursoy & Karali (2003 ▶); Borges *et al.* (2005 ▶). For the synthesis, see: Zhou *et al.* (2008 ▶).
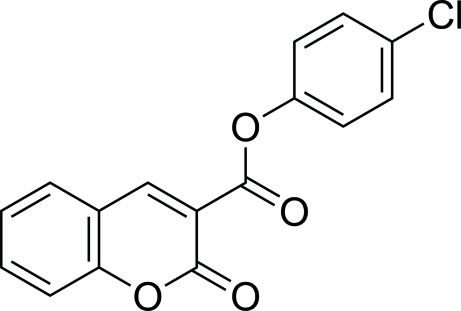


## Experimental

### 

#### Crystal data

C_16_H_9_ClO_4_
*M* *_r_* = 300.68
Monoclinic, 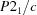

*a* = 15.7586 (6) Å
*b* = 7.0694 (2) Å
*c* = 12.7167 (6) Å
β = 113.037 (5)°
*V* = 1303.70 (9) Å^3^
*Z* = 4
Mo *K*α radiation
μ = 0.31 mm^−1^
*T* = 135 K
0.35 × 0.30 × 0.25 mm

#### Data collection

Agilent Xcalibur Eos diffractometer
Absorption correction: multi-scan (*CrysAlis PRO*; Agilent, 2011 ▶) *T*_min_ = 0.99, *T*_max_ = 1.00
5907 measured reflections
2662 independent reflections
2059 reflections with *I* > 2σ(*I*)
*R*_int_ = 0.025

#### Refinement

*R*[*F* ^2^ > 2σ(*F* ^2^)] = 0.040
*wR*(*F* ^2^) = 0.092
*S* = 1.04
2662 reflections
190 parameters
H-atom parameters constrained
Δρ_max_ = 0.22 e Å^−3^
Δρ_min_ = −0.25 e Å^−3^

### 

Data collection: *CrysAlis PRO* (Agilent, 2011 ▶); cell refinement: *CrysAlis PRO*; data reduction: *CrysAlis PRO*; program(s) used to solve structure: *SHELXTL* (Sheldrick, 2008 ▶); program(s) used to refine structure: *SHELXTL*; molecular graphics: *OLEX2* (Dolomanov *et al.*, 2009 ▶); software used to prepare material for publication: *OLEX2*.

## Supplementary Material

Crystal structure: contains datablock(s) global, I. DOI: 10.1107/S1600536812003376/xu5453sup1.cif

Structure factors: contains datablock(s) I. DOI: 10.1107/S1600536812003376/xu5453Isup2.hkl

Supplementary material file. DOI: 10.1107/S1600536812003376/xu5453Isup3.cml

Additional supplementary materials:  crystallographic information; 3D view; checkCIF report

## Figures and Tables

**Table 1:** Hydrogen-bond geometry (Å, °)

*D*—H⋯*A*	*D*—H	H⋯*A*	*D*⋯*A*	*D*—H⋯*A*
C3—H3⋯O2^i^	0.95	2.34	3.061 (2)	133

Symmetry code: (i) 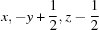.

**Fig. 1.**
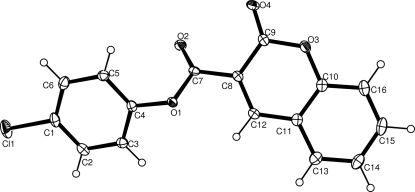
The molecular structure of the title compound with the atom numbering, showing displacement ellipsoids at the 30% probability level.

**Fig. 2.**
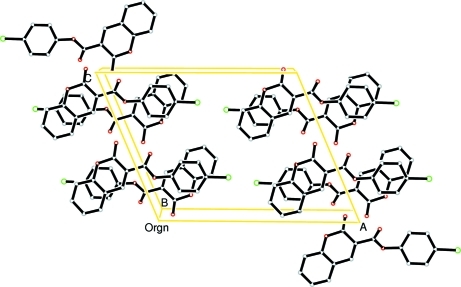
A packing diagram of the title compound.

## supplementary crystallographic information

### Comment

The coumarins and derivatives have demonstrated an ever-increasing variety of uses, including platelet anti-aggregating activity, anti-inflammatory activity (Kontogiorgis & Hadjipavlou-Litina, 2005), anti-tumor activity (Finn *et al.*, 2002), anti-bacterials (Gursoy & Karali, 2003), and antiviral effect (Borges *et al.*, 2005). So the title compound was synthesized according to the published method (Zhou *et al.*, 2008). We report here the crystal structure of the title compound. In the title compound (Fig. 1), the dihedral angle between the planes of coumarin and benzene ring is 22.60 (7)°. The packing view of the title compound is shown in Fig. 2, weak intermolecular C—H···O hydrogen bonding is present in the crystal (Table 1).

### Experimental

2-Oxo-2*H*-chromene-3-carboxylic acid (0.02 mol) was added to 10 ml sulfurous oxychloride. The mixture was refluxed for 3 h, and then the resultant was removed with simple distillation to give 2-oxo-2*H*-chromene-3-carbonyl chloride (3.95 g). The compound can be used directly without purification. The solution of 4-chlorophenol (0.0165 mol) dissolved in dried methyl dichloride (15 ml) was added dropwise to a solution of 2-oxo-2*H*-chromene-3-acyl chloride (0.015 mol) dissolved in methyl dichloride (20 ml) and triethylamine (2.5 ml) at room temperature. The reaction mixture was refluxed for 6 h, (mornitored by TLC). The mixture was then neutralized with 5% HCl and washed with saturated NaHCO_3_ and brine respectively. The organic phase was dried over Na_2_SO_4_ and evaporated under the reduced pressure. The resulting residue was purified by column chromatography (ethyl acetate: petroleum ether) to give the pure compound. Single crystals suitable for X-ray analysis were obtained by slow evaporation of a mixed solvent (methyl dichloride: methanol) at room temperature.

### Refinement

All H atoms were placed in calculated positions and refined in the riding model approximation, with C—H = 0.95 Å. The hydrogen atoms were refined in the riding model with *U*_iso_(H) = 1.2*U*_eq_(C).

### Crystal data

**Table:**

C_16_H_9_ClO_4_	*F*(000) = 616
*M**_r_* = 300.68	*D*_x_ = 1.532 Mg m^−^^3^
Monoclinic, *P*2_1_/*c*	Mo *K*α radiation, λ = 0.7107 Å
*a* = 15.7586 (6) Å	Cell parameters from 1865 reflections
*b* = 7.0694 (2) Å	θ = 3.2–29.0°
*c* = 12.7167 (6) Å	µ = 0.31 mm^−^^1^
β = 113.037 (5)°	*T* = 135 K
*V* = 1303.70 (9) Å^3^	Block, colorless
*Z* = 4	0.35 × 0.30 × 0.25 mm

### Data collection

**Table:**

Agilent Xcalibur Eos diffractometer	2662 independent reflections
Radiation source: Enhance (Mo) X-ray Source	2059 reflections with *I* > 2σ(*I*)
Graphite monochromator	*R*_int_ = 0.025
Detector resolution: 16.0874 pixels mm^-1^	θ_max_ = 26.4°, θ_min_ = 3.2°
ω scans	*h* = −19→13
Absorption correction: multi-scan (*CrysAlis PRO*; Agilent, 2011)	*k* = −8→8
*T*_min_ = 0.99, *T*_max_ = 1.00	*l* = −15→15
5907 measured reflections	

### Refinement

**Table:**

Refinement on *F*^2^	Primary atom site location: structure-invariant direct methods
Least-squares matrix: full	Secondary atom site location: difference Fourier map
*R*[*F*^2^ > 2σ(*F*^2^)] = 0.040	Hydrogen site location: inferred from neighbouring sites
*wR*(*F*^2^) = 0.092	H-atom parameters constrained
*S* = 1.04	*w* = 1/[σ^2^(*F*_o_^2^) + (0.0338*P*)^2^ + 0.3053*P*] where *P* = (*F*_o_^2^ + 2*F*_c_^2^)/3
2662 reflections	(Δ/σ)_max_ < 0.001
190 parameters	Δρ_max_ = 0.22 e Å^−^^3^
0 restraints	Δρ_min_ = −0.25 e Å^−^^3^

### Special details

**Table:**

Geometry. All e.s.d.'s (except the e.s.d. in the dihedral angle between two l.s. planes) are estimated using the full covariance matrix. The cell e.s.d.'s are taken into account individually in the estimation of e.s.d.'s in distances, angles and torsion angles; correlations between e.s.d.'s in cell parameters are only used when they are defined by crystal symmetry. An approximate (isotropic) treatment of cell e.s.d.'s is used for estimating e.s.d.'s involving l.s. planes.
Refinement. Refinement of *F*^2^ against ALL reflections. The weighted *R*-factor *wR* and goodness of fit *S* are based on *F*^2^, conventional *R*-factors *R* are based on *F*, with *F* set to zero for negative *F*^2^. The threshold expression of *F*^2^ > σ(*F*^2^) is used only for calculating *R*-factors(gt) *etc*. and is not relevant to the choice of reflections for refinement. *R*-factors based on *F*^2^ are statistically about twice as large as those based on *F*, and *R*- factors based on ALL data will be even larger.

### Fractional atomic coordinates and isotropic or equivalent isotropic displacement parameters (Å^2^)

**Table:**

	*x*	*y*	*z*	*U*_iso_*/*U*_eq_	
Cl1	0.43760 (4)	0.34411 (8)	0.78566 (6)	0.04706 (19)	
O1	0.06760 (8)	0.42742 (17)	0.78729 (10)	0.0207 (3)	
O2	0.08852 (9)	0.22946 (18)	0.93513 (11)	0.0261 (3)	
O3	−0.19477 (9)	0.33623 (17)	0.87422 (11)	0.0257 (3)	
O4	−0.05715 (9)	0.31077 (18)	1.01368 (11)	0.0282 (3)	
C1	0.32829 (13)	0.3605 (3)	0.78930 (18)	0.0278 (5)	
C2	0.25165 (13)	0.3316 (3)	0.69014 (17)	0.0268 (4)	
H2	0.2583	0.2995	0.6212	0.032*	
C3	0.16458 (13)	0.3502 (2)	0.69216 (16)	0.0224 (4)	
H3	0.1108	0.3305	0.6249	0.027*	
C4	0.15761 (12)	0.3978 (2)	0.79363 (15)	0.0198 (4)	
C5	0.23437 (13)	0.4279 (3)	0.89344 (15)	0.0233 (4)	
H5	0.2278	0.4620	0.9621	0.028*	
C6	0.32111 (13)	0.4072 (3)	0.89098 (17)	0.0281 (4)	
H6	0.3750	0.4249	0.9585	0.034*	
C7	0.03900 (13)	0.3290 (2)	0.85939 (15)	0.0194 (4)	
C8	−0.06155 (12)	0.3544 (2)	0.82490 (15)	0.0189 (4)	
C9	−0.09937 (13)	0.3309 (2)	0.91268 (16)	0.0216 (4)	
C10	−0.25169 (13)	0.3560 (2)	0.76084 (16)	0.0235 (4)	
C11	−0.21523 (13)	0.3850 (2)	0.67830 (16)	0.0218 (4)	
C12	−0.11740 (13)	0.3840 (2)	0.71464 (15)	0.0199 (4)	
H12	−0.0910	0.4045	0.6600	0.024*	
C13	−0.27666 (14)	0.4046 (3)	0.56409 (17)	0.0276 (4)	
H13	−0.2535	0.4265	0.5065	0.033*	
C14	−0.37008 (14)	0.3924 (3)	0.53509 (18)	0.0338 (5)	
H14	−0.4114	0.4052	0.4575	0.041*	
C15	−0.40409 (14)	0.3612 (3)	0.6193 (2)	0.0352 (5)	
H15	−0.4688	0.3515	0.5985	0.042*	
C16	−0.34558 (14)	0.3440 (3)	0.73263 (19)	0.0305 (5)	
H16	−0.3692	0.3243	0.7900	0.037*	

### Atomic displacement parameters (Å^2^)

**Table:**

	*U*^11^	*U*^22^	*U*^33^	*U*^12^	*U*^13^	*U*^23^
Cl1	0.0280 (3)	0.0510 (4)	0.0715 (4)	0.0092 (3)	0.0296 (3)	0.0160 (3)
O1	0.0195 (7)	0.0249 (7)	0.0200 (7)	0.0019 (6)	0.0102 (5)	0.0047 (5)
O2	0.0250 (7)	0.0309 (7)	0.0226 (7)	0.0051 (6)	0.0096 (6)	0.0066 (6)
O3	0.0240 (7)	0.0298 (7)	0.0276 (7)	−0.0018 (6)	0.0147 (6)	−0.0031 (6)
O4	0.0325 (8)	0.0361 (8)	0.0192 (7)	−0.0040 (7)	0.0134 (6)	−0.0015 (6)
C1	0.0216 (10)	0.0223 (10)	0.0436 (12)	0.0038 (8)	0.0173 (9)	0.0083 (9)
C2	0.0328 (11)	0.0231 (10)	0.0310 (11)	0.0017 (9)	0.0196 (9)	0.0028 (8)
C3	0.0259 (10)	0.0196 (9)	0.0216 (10)	−0.0023 (8)	0.0093 (8)	0.0005 (7)
C4	0.0201 (9)	0.0168 (8)	0.0244 (10)	0.0017 (8)	0.0107 (8)	0.0038 (7)
C5	0.0257 (10)	0.0230 (10)	0.0208 (10)	−0.0012 (8)	0.0086 (8)	0.0015 (7)
C6	0.0210 (10)	0.0264 (10)	0.0318 (11)	−0.0004 (9)	0.0049 (9)	0.0061 (8)
C7	0.0256 (10)	0.0195 (9)	0.0149 (9)	−0.0018 (8)	0.0099 (8)	−0.0030 (7)
C8	0.0222 (9)	0.0155 (9)	0.0222 (9)	−0.0010 (8)	0.0122 (8)	−0.0020 (7)
C9	0.0241 (10)	0.0180 (9)	0.0265 (11)	−0.0027 (8)	0.0140 (8)	−0.0034 (8)
C10	0.0228 (10)	0.0181 (9)	0.0297 (11)	−0.0006 (8)	0.0105 (8)	−0.0037 (8)
C11	0.0223 (10)	0.0167 (9)	0.0267 (10)	0.0012 (8)	0.0098 (8)	−0.0018 (7)
C12	0.0253 (10)	0.0156 (8)	0.0216 (9)	−0.0006 (8)	0.0123 (8)	−0.0017 (7)
C13	0.0285 (11)	0.0224 (10)	0.0292 (11)	0.0039 (9)	0.0083 (9)	0.0004 (8)
C14	0.0257 (11)	0.0289 (11)	0.0376 (12)	0.0066 (9)	0.0025 (9)	0.0002 (9)
C15	0.0192 (10)	0.0266 (11)	0.0554 (14)	0.0038 (9)	0.0098 (10)	−0.0021 (10)
C16	0.0249 (11)	0.0255 (10)	0.0450 (13)	0.0011 (9)	0.0178 (10)	−0.0019 (9)

### Geometric parameters (Å, º)

**Table:**

Cl1—C1	1.7450 (19)	C6—H6	0.9500
O1—C4	1.404 (2)	C7—C8	1.480 (2)
O1—C7	1.361 (2)	C8—C9	1.466 (2)
O2—C7	1.201 (2)	C8—C12	1.349 (2)
O3—C9	1.388 (2)	C10—C11	1.395 (3)
O3—C10	1.375 (2)	C10—C16	1.382 (3)
O4—C9	1.202 (2)	C11—C12	1.426 (3)
C1—C2	1.378 (3)	C11—C13	1.402 (3)
C1—C6	1.381 (3)	C12—H12	0.9500
C2—H2	0.9500	C13—H13	0.9500
C2—C3	1.388 (3)	C13—C14	1.373 (3)
C3—H3	0.9500	C14—H14	0.9500
C3—C4	1.379 (2)	C14—C15	1.390 (3)
C4—C5	1.385 (2)	C15—H15	0.9500
C5—H5	0.9500	C15—C16	1.379 (3)
C5—C6	1.387 (3)	C16—H16	0.9500
			
C7—O1—C4	118.58 (13)	C12—C8—C9	120.99 (17)
C10—O3—C9	122.99 (14)	O3—C9—C8	115.83 (16)
C2—C1—Cl1	119.13 (16)	O4—C9—O3	116.77 (16)
C2—C1—C6	121.90 (18)	O4—C9—C8	127.39 (18)
C6—C1—Cl1	118.94 (16)	O3—C10—C11	120.82 (17)
C1—C2—H2	120.4	O3—C10—C16	117.30 (17)
C1—C2—C3	119.20 (18)	C16—C10—C11	121.88 (19)
C3—C2—H2	120.4	C10—C11—C12	117.83 (17)
C2—C3—H3	120.6	C10—C11—C13	118.25 (18)
C4—C3—C2	118.79 (17)	C13—C11—C12	123.85 (18)
C4—C3—H3	120.6	C8—C12—C11	121.38 (17)
C3—C4—O1	115.54 (16)	C8—C12—H12	119.3
C3—C4—C5	122.29 (17)	C11—C12—H12	119.3
C5—C4—O1	122.01 (16)	C11—C13—H13	119.8
C4—C5—H5	120.7	C14—C13—C11	120.33 (19)
C4—C5—C6	118.56 (17)	C14—C13—H13	119.8
C6—C5—H5	120.7	C13—C14—H14	120.0
C1—C6—C5	119.26 (18)	C13—C14—C15	119.9 (2)
C1—C6—H6	120.4	C15—C14—H14	120.0
C5—C6—H6	120.4	C14—C15—H15	119.4
O1—C7—C8	109.68 (15)	C16—C15—C14	121.15 (19)
O2—C7—O1	123.86 (17)	C16—C15—H15	119.4
O2—C7—C8	126.34 (17)	C10—C16—H16	120.8
C9—C8—C7	117.88 (16)	C15—C16—C10	118.43 (19)
C12—C8—C7	120.95 (16)	C15—C16—H16	120.8
			
Cl1—C1—C2—C3	−178.09 (13)	C7—C8—C9—O3	−173.49 (15)
Cl1—C1—C6—C5	177.43 (14)	C7—C8—C9—O4	7.9 (3)
O1—C4—C5—C6	−175.94 (16)	C7—C8—C12—C11	172.06 (16)
O1—C7—C8—C9	−154.96 (14)	C9—O3—C10—C11	−4.4 (2)
O1—C7—C8—C12	29.9 (2)	C9—O3—C10—C16	174.89 (16)
O2—C7—C8—C9	28.8 (3)	C9—C8—C12—C11	−2.9 (3)
O2—C7—C8—C12	−146.26 (18)	C10—O3—C9—O4	−179.14 (15)
O3—C10—C11—C12	3.0 (2)	C10—O3—C9—C8	2.1 (2)
O3—C10—C11—C13	−179.87 (15)	C10—C11—C12—C8	0.6 (3)
O3—C10—C16—C15	−179.25 (16)	C10—C11—C13—C14	−1.0 (3)
C1—C2—C3—C4	0.3 (3)	C11—C10—C16—C15	0.1 (3)
C2—C1—C6—C5	−0.8 (3)	C11—C13—C14—C15	0.3 (3)
C2—C3—C4—O1	175.57 (15)	C12—C8—C9—O3	1.6 (2)
C2—C3—C4—C5	0.1 (3)	C12—C8—C9—O4	−177.06 (18)
C3—C4—C5—C6	−0.7 (3)	C12—C11—C13—C14	175.89 (18)
C4—O1—C7—O2	6.5 (2)	C13—C11—C12—C8	−176.34 (16)
C4—O1—C7—C8	−169.80 (14)	C13—C14—C15—C16	0.7 (3)
C4—C5—C6—C1	1.0 (3)	C14—C15—C16—C10	−0.8 (3)
C6—C1—C2—C3	0.1 (3)	C16—C10—C11—C12	−176.25 (17)
C7—O1—C4—C3	126.23 (16)	C16—C10—C11—C13	0.8 (3)
C7—O1—C4—C5	−58.3 (2)		

### Hydrogen-bond geometry (Å, º)

**Table:**

*D*—H···*A*	*D*—H	H···*A*	*D*···*A*	*D*—H···*A*
C3—H3···O2^i^	0.95	2.34	3.061 (2)	133

Symmetry code: (i) *x*, −*y*+1/2, *z*−1/2.
